# Drug-Releasing Antibacterial Coating Made from Nano-Hydroxyapatite Using the Sonocoating Method

**DOI:** 10.3390/nano11071690

**Published:** 2021-06-28

**Authors:** Khaled AbouAitah, Monika Bil, Elzbieta Pietrzykowska, Urszula Szałaj, Damian Fudala, Bartosz Woźniak, Justyna Nasiłowska, Anna Swiderska-Sroda, Maciej Lojkowski, Barbara Sokołowska, Wojciech Swieszkowski, Witold Lojkowski

**Affiliations:** 1 Laboratory of Nanostructures and Nanomedicine, Institute of High Pressure Physics, Polish Academy of Sciences, 29/37 Sokolowska Street, 01142 Warsaw, Poland; e.pietrzykowska@labnano.pl (E.P.); u.szalaj@labnano.pl (U.S.); d.fudala@labnano.pl (D.F.); wozniakbartoszz@gmail.com (B.W.); a.swiderska-sroda@labnano.pl (A.S.-S.); 2 Medicinal and Aromatic Plants Research Department, Pharmaceutical and Drug Industries Research Division, National Research Centre (NRC), Dokki, Giza 12622, Egypt; 3 Centre for Advanced Materials and Technologies, Warsaw University of Technology, Poleczki 19, 02822 Warsaw, Poland; monika.bil@pw.edu.pl; 4 Faculty of Materials Science and Engineering, Warsaw University of Technology, 141 Woloska Street, 02507 Warsaw, Poland; Maciej.Lojkowski.dokt@pw.edu.pl (M.L.); wojciech.swieszkowski@pw.edu.pl (W.S.); 5 Department of Microbiology, Prof. Wacław Dąbrowski Institute of Agriculture and Food Biotechnology–State Research Institute, 36 Rakowiecka Street, 02532 Warsaw, Poland; justyna.nasilowska@ibprs.pl (J.N.); barbara.sokolowska@ibprs.pl (B.S.); 6 High Pressure Food and Soft Matter Processing Group, Institute of High-Pressure Physics, Polish Academy of Sciences, 29/37 Sokołowska Street, 01142 Warsaw, Poland

**Keywords:** antibacterial coating, hydroxyapatite nanoparticles, sonocoating, PEEK implants, cefuroxime sodium salt antibiotic, drug release, *Staphylococcus aureus* bacteria

## Abstract

Medical implant use is associated with a risk of infection caused by bacteria on their surface. Implants with a surface that has both bone growth-promoting properties and antibacterial properties are of interest in orthopedics. In the current study, we fabricated a bioactive coating of hydroxyapatite nanoparticles on polyether ether ketone (PEEK) using the sonocoating method. The sonocoating method creates a layer by immersing the object in a suspension of nanoparticles in water and applying a high-power ultrasound. We show that the simple layer fabrication method results in a well-adhering layer with a thickness of 219 nm to 764 nm. Dropping cefuroxime sodium salt (Cef) antibiotic on the coated substrate creates a layer with a drug release effect and antibacterial activity against *Staphylococcus aureus*. We achieved a concentration of up to 1 mg of drug per cm^2^ of the coated substrate. In drug release tests, an initial burst was observed within 24 h, accompanied by a linear stable release effect. The drug-loaded implants exhibited sufficient activity against S. *aureus* for 24 and 168 h. Thus, the simple method we present here produces a biocompatible coating that can be soaked with antibiotics for antibacterial properties and can be used for a range of medical implants.

## 1. Introduction

Coating the surface of medical implants to provide biocompatibility, bioactivity, antibacterial, anticancer, and bone-growth properties is a well-established technology [1]. Implantation leads to a risk of infection caused by bacteria attached to the implant surface. The risk is particularly high for patients with low systemic and local immunity, as these bacteria may invade the circulation or remain in the tissues surrounding the inserted implant [2]. Among other bacteria, *Staphylococcus*, *Enterobacteriaceae,* and *Pseudomonas* cause considerable medical problems because they can attach to the implant surface, producing a biofilm [3]. In the case of infection with pathogens such as the Gram-positive *Staphylococcus aureus*, site infections occur in >5% of medical implant and device implantations [4]. The critical time of colony formation by bacteria after the placement of any implant is from a few hours up to 72 h [5]. Many studies have shown that the stage of bacterial attachment and biofilm formation lasts from 12 to 24 h, and proliferation and maturation of the biofilm occurs from 36 to 72 h [5,6]. After the biofilm is established, bacterial cells can spread in the tissues, causing chronic infections. Thus, preventing biofilm formation is essential to reducing the risk of post-operative infection [6]. Consequently, there are different strategies toward bacterial biofilm, including surface modification of medical devices/implants [7], small molecule biofilm inhibitors [8], biofilm dispersal agents [9], and antibacterial coatings [10,11]. Antibacterial coatings may prevent biofilm formation and prevent inflammation [2,12] and are employed to locally deliver antibiotic drugs and therapeutic agents for short- (few hours or days) or long-acting antibacterial effects (days and weeks) [13].

In this context, phosphate materials, especially hydroxyapatite phases, have been considered as coating materials for bone implants because they are similar to the mineral components of the bone tissue and their biocompatibility [14,15,16]. Micro and nanoparticles of hydroxyapatite (nanoHAP) have been used for coatings to enhance bone formation and in drug delivery systems to carry drugs and deliver growth factors and proteins [16,17,18,19,20,21]. The local delivery and release of drugs by a nanoHAP coating permits highly efficient targeting of the infected area with reduced cytotoxicity [18,19,22]. The layer properties may strongly depend on such properties of the synthesized particles as specific surface area and particle size. For example, Szalaj, et al. demonstrated that nanoparticle size strongly changes the kinetics and efficiency of water adsorption, which is related to the specific surface area [23]. Several attempts to achieve long-term antibacterial actions using antibiotic-loaded HAP [18,20,24] or HAP with antibacterial doping [25,26] have been reported. However, a coating with short-term antibacterial action that enhances subsequent bone growth is still a challenge.

Polyether ether ketone (PEEK) is a candidate material for implants because of its chemical resistance and its mechanical properties matching that of the bone and radiolucency [21,27]. PEEK possesses an elastic modulus (3.2 GPa) between that of cortical (15–30 GPa) and cancellous bone (0.5–1.5 GPa) [21]. In addition, PEEK has a bio-inert and hydrophobic nature, which results in poor osseointegration characteristics [27,28]. Therefore, coatings and surface modifications have been developed that improve its bioactive properties and increase its osseointegration properties. One of these techniques is to coat the PEEK with hydroxyapatite.

**Scheme 1:**
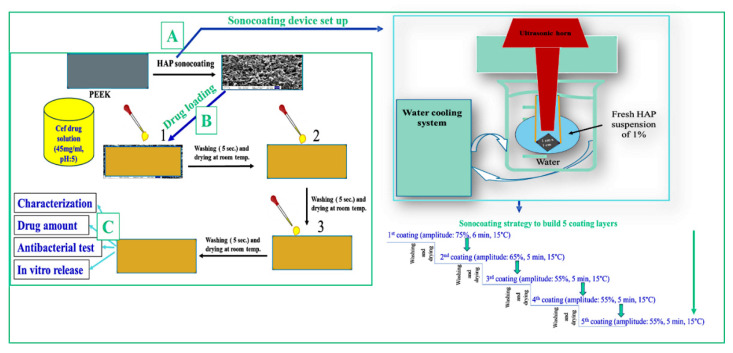
Schematic of controlled nanolayer coating and antibacterial evaluation. (**A**) Preparation method for one to five iterations of coating to obtain a nanolayer thickness using optimized parameters. (**B**) Drug loading method. (**C**) Characterized materials and their antibacterial effects.

Several methods have been developed for coating PEEK with hydroxyapatite [29,30,31]. Johansson, et al. [21] reported nano-sized coating of hydroxyapatite crystal on PEEK implants to enhance bone formation. However, this method requires many steps and high temperature, and there is an organic residue. Other methods include electrostatic and covalent attachment [32], electrophoretic deposition [18], electrochemical deposition [33], biomimetic precipitation [34], physical vapor deposition techniques [35,36], and plasma spraying [37], among others. However, the coatings obtained using these methods have a thickness measured in micrometers and a non-porous structure. Thus, another technique is required to produce thin nanoscale layers with a porous structure able to carry the antibacterial drugs. In the last decade, a sonochemical technique has been developed for creating antibacterial coatings of metal nanoparticles on different surfaces, mostly textiles for medical applications [38,39,40,41]. We attempted to use this method to obtain a nanoHAP coating on the PEEK surface. To coat the object, it is immersed in a nanoparticle suspension in water. Treatment of the suspension with high-power ultrasound waves leads to attachment of the nanoparticles to the surface of the object. This is a green chemistry approach as no chemicals are used. The advantage of this method is that it is a single, easy step at low cost and low temperature. The mechanism by which the nanoHAP layer is created on a polymer was recently described by Wozniak, et al. [42]. The nanoHAP layer(s) is grown laterally in a dendrite-like pattern on the surface. Sonocoating with nanoHAP on bone regrowth scaffolds has also been shown to enhance bone regeneration [16]. Therefore, sonocoating is considered an excellent approach for obtaining nanoHAP layers on various substrates by means of green chemistry. Therefore, in the current study, we sonocoated nanoHAP onto PEEK substrates to create a biocompatible layer (Scheme 1) with a potential to stimulate bone growth while absorbing antibacterial drugs. We applied antibiotic cefuroxime sodium salt (Cef) and carried out drug release tests. We selected Cef as a model antibiotic, a second-generation cephalosporin, because it is efficient and has broad applications against both Gram-negative and Gram-positive micro-organisms [43,44,45]. The sodium salt form of Cef has been used for a long time due to its solubility, injection application, various purposes in clinical practice, and sparse drug allergy in patients [46,47]. For these reasons, it is frequently used as a prophylaxis in total joint replacement and an antibiotic in orthopedic surgery [48,49,50].

## 2. Materials and Methods

### 2.1. Synthesis of nanoHAP Solutions for Coating

GoHAP™, developed at the Institute of High Pressure Physics, Polish Academy of Sciences (IHPP PAS), were the nanoHAP used for sonocoating. GoHAP1 and GoHAP3 were selected because they exhibit different particle sizes and specific surface areas. The procedure has been described in detail elsewhere [16,23,51]. GoHAP1 particles (approximately 9 nm in size with a specific surface area of 206 ± 1 m^2^/g) and GoHAP3 particles (approximately 16 nm in size with a specific surface area 149 ± 1 m^2^/g) were synthesized using the MSS2 microwave reactor (manufactured by IHPP PAS in collaboration with Łukasiewicz Research Network—The Institute for Sustainable Technologies, Radom, Poand) at 3 kW and 2.45 GHz [52].

### 2.2. Sonocoating of PEEK Implants

The PEEK medical implant substrate (Goodfellow Cambridge Limited, Huntingdon, UK) was cut into 1 cm^2^ plates and cleaned in ethanol for 15 min using a water bath sonicator (Elma water bath sonicator, Singen, Germany). For sonocoating, we used suspensions of the two different types of GoHAP™ at a concentration of 1 wt % in distilled water. Coating was performed in subsequent steps for one to five iterations under controlled conditions as shown in Scheme 1A. The sonocoating process used a Ø 13 mm diameter titanium ultrasonic horn at 20 kHz frequency. The VCX 750 generator (Sonics, Newtown, CT, USA), operating at 750 W, provided ultrasonic energy. During coating, a 75% vibration amplitude was applied for 6 min, and then the PEEK substrate was washed in deionized water for 5 s under magnetic stirring. The samples were left at room temperature to dry. To increase the layer thickness, the coating procedure was repeated with smaller amplitudes and shorter times (second cycle: 65% and 5 min; third to fifth cycle: 55% for 5 min). For each iteration, a fresh suspension was prepared. Samples were dried between the coatings.

### 2.3. Incorporating the Antibacterial Drug in Sonocoated nanoHAP

Geuli, et al. [18] first loaded antibiotic drugs on HAP nanoparticles and then fabricated the antibacterial coating. However, this technique may affect the chemical structure of the used drugs (e.g., use of high temperature for fabrication process, strong solvents, etc.). Here, we used the simple drop-by-drop method to saturate the coated surface. The drug solution (45 mg/mL) was initially adjusted to an acidic pH of 5. The prepared solution was applied by dropping the equivalent of 50 µL onto the surface of the dried GoHAP-coated samples, which were then washed with deionized water under medium stirring (300 rpm) for 5 s and left at room temperature to dry. The procedure was repeated three times. The obtained samples were named PEEK-HAP1-Cef and PEEK-HAP3-Cef.

### 2.4. Determination of the Amount of Loaded Drug

To measure the drug loading, we extracted the loaded drug by placing the samples in a glass bottle containing deionized water (3 mL) and sonicating it for 15 min (Elma water bath sonicator, Singen, Germany). The solution was collected, and the procedure repeated a second time to ensure that all drug was extracted. Next, the collected sonicated solution (6 mL) was filtered using a syringe filter (0.45 µm) to remove any nanoHAP particles that could influence the UV–Vis measurements. The quantative determination was performed by measuring the samples at 275 nm on an Evolution™ 60 UV–Visible spectrophotometer (Thermo Scientific, Pittsburgh, PA, USA) using a standard curve prepared for Cef. Each measurement was replicated five times.

### 2.5. In Vitro Drug Release Study

The PEEK-HAP1-Cef and PEEK-HAP3-Cef samples were placed separately in glass bottles with a screw cap and 3 mL of phosphate-buffered saline (PBS) buffer was added. Next, the samples were placed in an immersion circulator with a submersible multi-position stirrer at 37 °C and 150 rpm (IKA Poland, Warsaw, Poland). At specified time points (from 2 h to 150 h), the entire volume of PBS was sampled and stored in vials at 4 °C until UV–Vis analysis. The removed sample was replaced with a fresh release medium. The solutions were measured by using the Evolution™ 60 UV–Visible Spectrophotometer at 275 nm. The Cef concentration at each time point was determined based on the previously prepared calibration curve. Three measurements were performed for each sample. The cumulative drug release was calculated based on the following equations:

% of mass released at time *t* = (mass(*t*)/total mass of Cef in the coated substrate) × 100 (1)

cumulative release % = mass (*t* − 1) + mass (*t*) for current measurement (2)

where *t* is the percentage release at the time of measurement and *t* − 1 is the percentage of released drug at the previous time point.

### 2.6. Characterization Techniques

Several characterization techniques were used to investigate the nanolayer. Scanning electron microscopy (SEM) was performed using an Ultra Plus Scanning Electron Microscope (Zeiss, Jena, Germany) to observe the morphology of the layer (s) of nanoHAP on the surface and the thickness of the coated layer (s). The coated PEEK pellets were fractured in liquid nitrogen. The coat thickness was determined from SEM images using the Gwyddion software (version 2.28, Brno, Czech Republic) [42]. The pore size distribution of the coatings was analyzed in an Excel calculation program after SEM imaging. Fourier transform infrared (FTIR) spectroscopy was performed to determine the functional groups before and after nanoHAP coating and drug loading. The Bruker Optics Tensor 27 FTIR instrument (Bruker Corporation, Billerica, MA, USA) was equipped with an attenuated total reflectance (Platinum ATR-Einheit A 255). For surface topography, atomic force microscopy (AFM) images were obtained by MFP-3D-Bio (Asylum Research, Oxford Instruments, High Wycombe, UK). We tested the wetting angle using an OCA 20 goniometer (DataPhysics Instruments GmbH, Filderstadt, Germany), and measured the contact angle by applying a drop of deionized water and immediately measuring.

### 2.7. Assessment of Antimicrobial Activity

The antimicrobial activity was determined by the inhibition zone method and evaluated against *S. aureus* ATCC 25923. In a typical experiment, 100 µL of the overnight culture (~10^8^ cfu/mL) in Tryptone Soy Broth (TSB; Biokar Diagnostics, Beauvais, France) was spread onto Tryptone Soy Agar (TSA; Biokar Diagnostics, Beauvais, France). The coated PEEK-HAP1 containing Cef was placed on the prepared agar plates and incubated at 37 °C. A plate with only *S. aureus* ATCC 25923 and a plate with *S. aureus* ATCC 25923 and PEEK-HAP1 without antibiotic were incubated as controls. The inhibition zones were measured every 24 h for 11 days. Studies were performed in triplicate.

Broth dilution tests were carried out in TSB by measuring the optical density (OD) at 600 nm using NanoDrop ND-1000 UV-Vis (NanoDrop Technologies, Wilmington, DE, USA). For this purpose, 10 µL of the overnight culture of *S. aureus* ATCC 25923 in TSB was transferred to 3 mL of new TSB with PEEK-HAP1 and PEEK-HAP1-Cef. As a control, TSB with only *S. aureus* ATCC 25923 was incubated. For all samples, incubation was 37 °C for 7 days. All experiments were performed in triplicate.

For the broth dilution method with changing broth, 10 µL of the overnight culture of *S. aureus* ATCC 25923 in TSB was transferred to 3 mL of new TSB with PEEK-HAP-Cef. TSB with only *S. aureus* ATCC 25923, or *S. aureus* ATCC 25923 and PEEK-HAP1 were incubated as controls. All samples were incubated for 10 h at 37 °C. After measuring the OD, the broth was poured out and the samples washed with 3 mL of PBS (POCh, Gliwice, Poland). We then added new 3 mL portions of TSB with 10 µL *S. aureus* ATCC 25923. The actions were repeated after another 14 and 24 h. Studies were performed in triplicate.

### 2.8. Statistical Analysis

Significant differences were evaluated by one-way analysis of variance (ANOVA) with the least significant difference at *p* < 0.05 [53].

## 3. Results and Discussion

### 3.1. Microscopic Characterization

**Figure 1:**
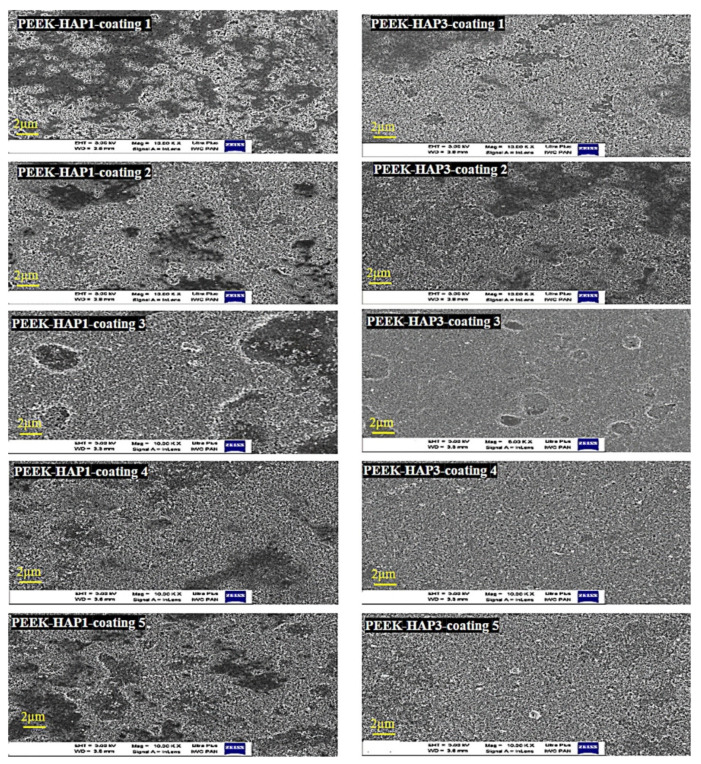
FE-SEM of the morphology of the GoHAP1 and GoHAP3 nanolayers on PEEK in five iterations.

**Figure 2:**
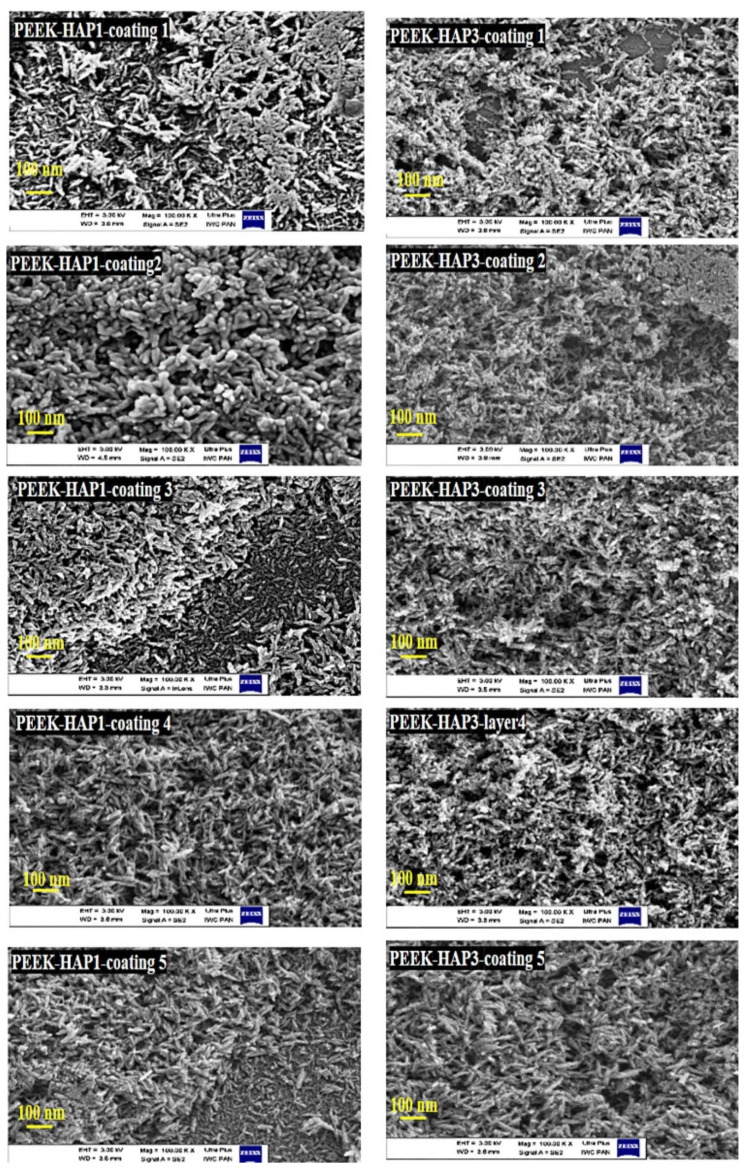
High-magnification FE-SEM images of the morphology of nanoHAP1 and nanoHAP3 after sonocoating PEEK in five subsequent iterations.

Figure 1 shows the coatings created with GoHAP1 and GoHAP3 on PEEK. The images show changes in the layer structure with each coating iteration for both GoHAP1 and GoHAP3. Using a high amplitude at the beginning and a low amplitude with shorter time in the next iteration (Scheme 1A) prevented damage and cracking of the layers, achieving a thickness of up to 700 nm. It is clear that the creation of the first, second, and third layers was associated with the appearance of some “islands”, whereas more even coverage was observed with the fourth and fifth coatings (especially when observed by the in-lens detector on the left). The coating thickness increased from the first to fifth layer. It seems that coating with GoHAP3 led to a more homogeneous layer compared to GoHAP1. In the latter case, a higher tendency to form islands was observed. In addition, the GoHAP layer had a porous structure when observed by the SE2 detector (Figure 2).

**Figure 3:**
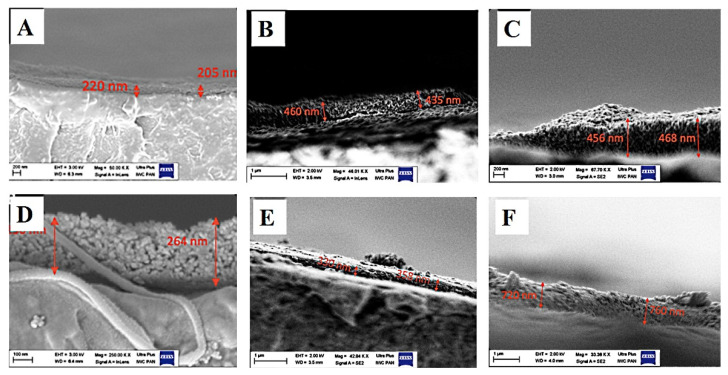
The thickness of layers after sonocoating PEEK substrate with GoHAP1 (**A**–**C**) and GoHAP3 (**D**–**F**). (**A**) GoHAP1 for one iteration, (**B**) three iterations, and (**C**) five iterations. (**D**) GoHAP3 for one iteration, (**E**) two iterations and (**F**) five iterations.

Images of fracture-coated PEEK plates are shown in Figure 3. The images of the fractured coatings allowed measurement of the thickness (Table 1). The results illustrate the increase in the GoHAP1 layer thickness with each iteration. The coating was homogeneous, and no cracks were observed. Although the same number of layers was inserted with GoHAP1 and GoHAP3, the layers were proportionally thicker with GoHAP3. This may be due to the bigger grain size of GoHAP3 than GoHAP1.

Most techniques in the literature produce microscale HAP coatings using nanoHAP [18,33,54,55,56]. These approaches include thermal spraying, plasma exposure, surface modification, spin-coating, cold spraying, aerosol deposition, and sputtering. However, only a few studies have demonstrated the possibility of preparing thin HAP coatings with a thickness in the nano-range. For example, Johansson, et al. produced a thin film layer (20–40 nm) of HAP nanoparticles on PEEK using a deposition technique [21]. This deposited layer seems to not be homogeneous and does not have porosity like the coating obtained by our technique. Therefore, the use of sonochemistry as a green approach for forming bioactive coatings for implants is very promising compared to other traditional techniques, as they are complex processes and require specific conditions, such as high temperature.

**Figure 4:**
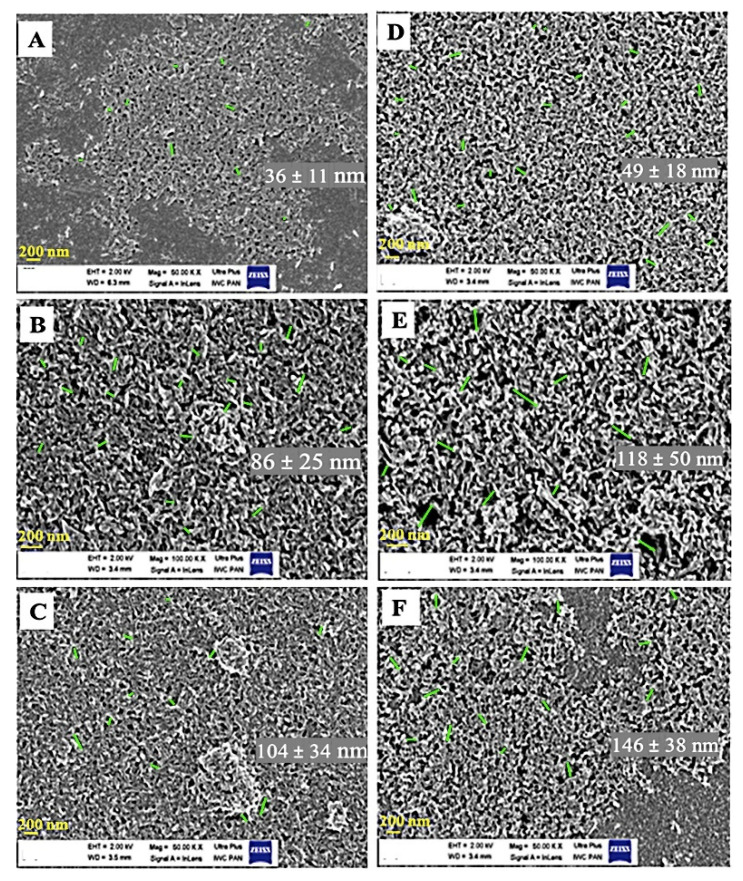
FE-SEM images showing the porosity of the sonocoated PEEK substrate in the nanometer range. (**A**) GoHAP1 for two iterations, (**B**) three iterations, and (**C**) five iterations. (**D**) GoHAP3 for two iterations, (**E**) three iterations and (**F**) five iterations. The green line shows the pore in the coating.

The porosity of the coatings is important for drug absorption. The porous structure of the HAP layer influences the bone implant interface, enhancing the ability to encapsulate drugs, induce osteogenic properties, and generate new bone formation [16,57,58]. FE-SEM images (Figure 4) showed that the obtained coatings had different nano-porous structures depending on the iteration of sonocoating. The porosity increased with the number of sonocoating iterations compared to a single layer. The pore size reached 104 ± 34 nm for PEEK-HAP1 and 146 ± 38 nm for PEEK-HAP3 in five iterations. Increased porosity can allow higher drug adsorption.

Overall, the characteristics of the prepared coatings met the requirements for improving drug loading, release and cell regeneration effects [59,60,61,62].

### 3.2. Contact Angle Measurement of Coatings

Table 2 shows the changes in contact angle before and after PEEK was coated with GoHAP. The GoHAP coatings had a significantly (*p* < 0.05) reduced contact angle with water compared to pristine PEEK (90° ± 1°). These results are in agreement with previous reports [16,63,64,65]. Comparing all iterations, the lowest contact angle (55° ± 2.3°) was recorded for PEEK-HAP3 coated three times. We also found a significant difference between coating with both GoHAP types. The contact angle was lower when coating with GoHAP3 compared to GoHAP1. The results are important for biomedical applications, as the reduced water contact angle contributes to improved cell attachment, bone growth and bioactivity [66,67,68].

### 3.3. AFM Analysis

**Figure 5:**
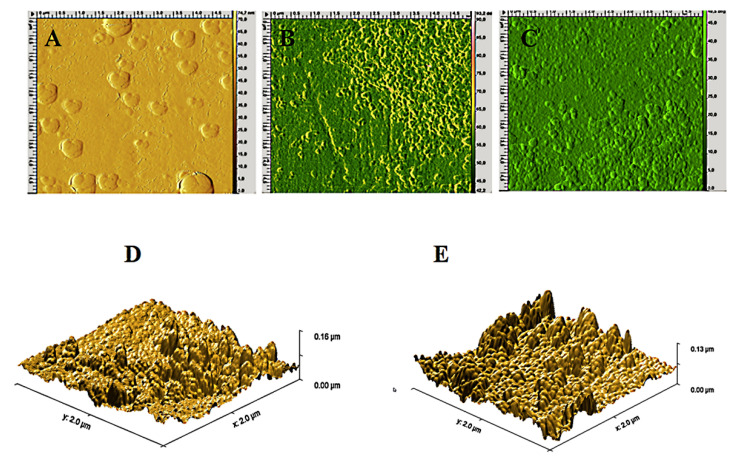
AFM images of uncoated and GoHAP1-coated PEEK after a single iteration. (**A**) Phase contrast of PEEK, (**B**) phase contrast of PEEK-GoHAP1, and (**C**) phase contrast of PEEK-GoHAP3. All are top views. (**D**) 3D projections of AFM images of PEEK-GoHAP1 and (**E**) PEEK-GoHAP3.

We employed AFM for surface imaging because it enables better three-dimensional imaging compared to SEM [69]. Sonocoatings prepared after one iteration of were imaged. Figure 5 shows that the coating on PEEK-GoHAP3 was more homogeneous than PEEK-GoHAP1 after the first coating (Figure 5B,C). Considering the topography of the surface, AFM images of both kinds of coating clearly indicated that the surface is not smooth. We detected that GoHAP nanoparticles formed vertical rod-like shapes (Figure 5D,E). The results are in line with a previous study from Li, et al. on a HAP-coated implant [70]. The topography of the coated surface may influence interfacial cellular functions and cell adhesion, eventually leading to strengthened biological actions between the surface and cells [70,71]. Another reason the sharp rodlike coating on the surface is important, is that it facilities the interaction with bacterial cells, such as those of *S. aureus* biofilm, and kills them.

### 3.4. FTIR Characterization

**Figure 6:**
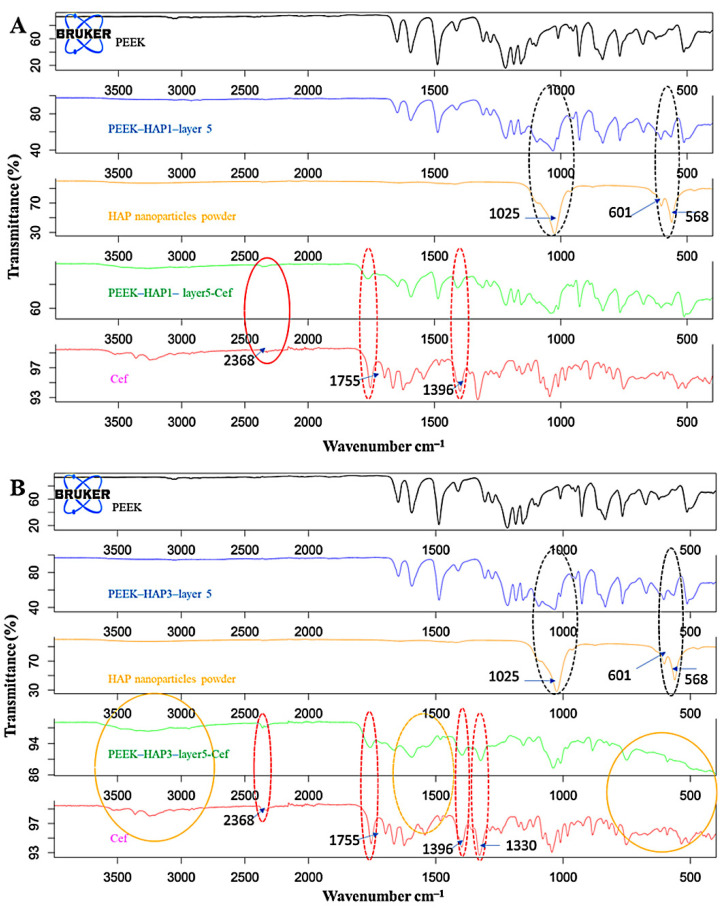
FTIR characterization of uncoated, coated, and Cef-loaded particles after five iterations of sonocoating. (**A**) GoHAP1, (**B**) GoHAP3.

The FTIR spectra of PEEK coated with GoHAP and loaded antibiotic are shown in Figure 6. Several peaks appeared for PEEK between 1500 and 500 cm^−1^, in line with previous studies [29,72]. Further coating with GoHAP resulted in new peaks centered at 1025, 601 and 586 cm^−1^, corresponding to the nanoHAP powder spectrum. These peaks are associated with the phosphate stretching of P–O at 1025 cm^−1^ and the bending of P–O–P at 601 and 586 cm^−1^ [73,74]. By adding the drug, several peaks corresponding to it can be seen in the PEEK-HAP spectra. In the case of PEEK-HAP1-Cef (Figure 6A), some peaks had increased intensity compared to PEEK-HAP1 (2368, 1755 and 1396 cm^−1^), whereas many new peaks were detected in the region of 1600 to 500 cm^−1^, indicating the presence of the drug. In the case of PEEK-HAP3-Cef (Figure 6B), some peaks had more intense peaks at 2368, 1755, 1396 and 1330 cm^−1^. A clear difference in the PEEK-HAP3-Cef spectrum compared to PEEK-HAP3, related to the presence of free Cef, was also seen in many regions: the 3500 to 3000 cm^−1^ region centered at ~1600 cm^−1^, and from approximately 700 to 500 cm^−1^. Several new peaks corresponding to Cef were also detected, reflecting the successful preparation and loading of Cef onto the nanoHAP coating.

### 3.5. Drug Loading and In Vitro Release

The results of the drug loading experiments are shown in Table 3. We found no significant difference between the amount of drug loaded with the two types of GoHAP. According to previous reports, the amount of drug is sufficient to achieve therapeutic efficacy. For example, Guillot, et al. [75] reported that the loading of bone morphogenetic proteins (BMPs) on PEEK reached 11.3 ± 0.3 µg/cm^2^. Another study by Min, et al. [76] on delivering gentamicin formed layer-by-layer reported 7 µg/cm^2^. Therefore, we hypothesize that such an amount would be sufficient to prevent biofilm formation.

**Figure 7:**
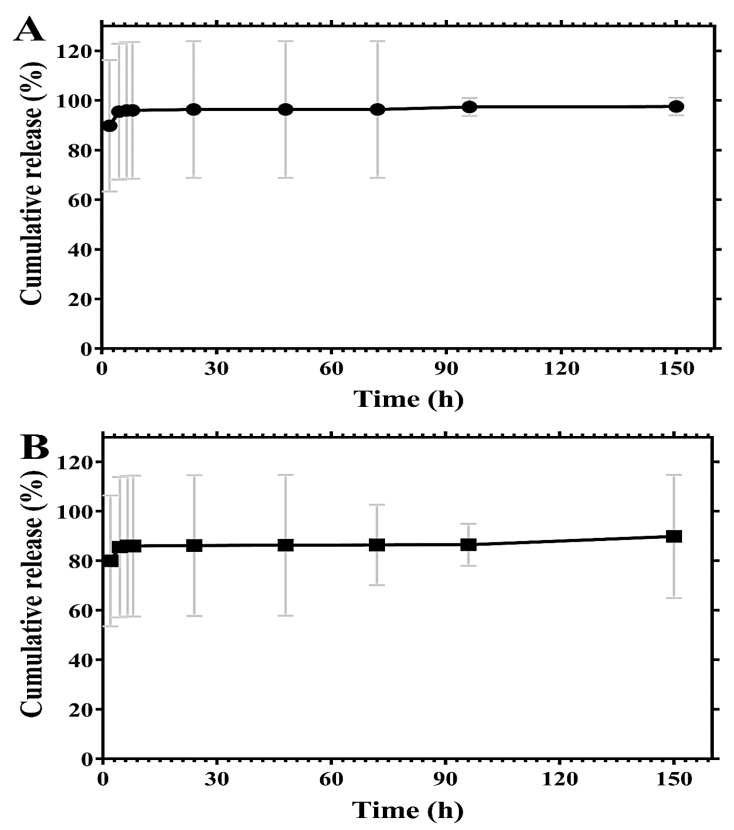
(**A**) In vitro release of Cef from PEEK-GoHAP1-Cef (**B**) and PEEK-GoHAP3-Cef in PBS release medium. Data are presented as mean ± SD.

The in vitro release profiles of Cef from GoHAP-coated PEEK are shown in Figure 7. There was a substantial initial “burst” release with nearly 96% of the drug being released in the first 24 h. The rapid release of Cef could be attributed to drug molecules attached to the surface having weak bonds and the high amount to the high porosity of the layer. A similar effect was observed for Cef released from PEEK coated with GoHAP3 but with a different release percentage (Figure 7B). Approximately 80%, 86% and 86.1% of the total amount of Cef was released within 2 h, 6.5 h and 24 h, respectively, followed by the release of approximately 86% up to 100 h, increasing to ~89% at 150 h. The quick release of Cef within 24 h accompanied by a linear effect up to 150 h is promising for the treatment of surgical site infection because *S. aureus* is able to produce biofilm between 24 and 72 h [5]. Therefore, such a release profile would be efficient to prevent attachment of the microbes. In contrast to cefuroxime release studies [77,78,79,80], no study has related the release profile of Cef sodium salt from nanoHAP or medical implants. The usage of Cef in sodium salt form by means of a local delivery system [46] is needed because the salt form is effective but not chemically stable [43].

### 3.6. Antibacterial Evaluations

**Figure 8:**
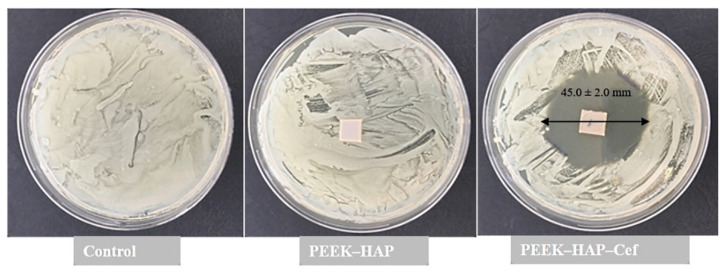
The antibacterial activity against *Staphylococcus aureus* by means of zone inhibition after incubation for 24 h with PEEK-GoHAP1 and PEEK-GoHAP1-Cef compared to control.

We used two different methods to evaluate the killing efficiency of the drug-infiltrated coatings against *S.* *aureus*. Figure 8 discloses the effective killing of *S. aureus* when grown on PEEK-HAP1-Cef compared to PEEK and PEEK-HAP1 in the inhibition zone test. The average inhibition zone diameter after 24 h was 45.0 mm ± 2.0 mm, which is considered a wide inhibition zone for the cultured bacteria. We observed that the zones were the same after 5 days of incubation and 11 days of incubation. These results are in line with previous studies on antibiotic coatings against *S. aureus* and other bacteria [17,18,81].

**Figure 9:**
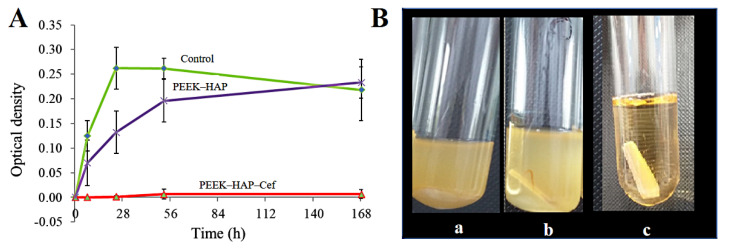
(**A**) The antibacterial activity against *Staphylococcus aureus* ATCC 25923 by means of the broth dilution method with PEEK-GoHAP1 and PEEK-GoHAP1-Cef compared to control. Data are presented as mean ± SD. (**B**) Photograph of the tubes after experiments: control (**a**), PEEK-GoHAP1 (**b**) and PEEK-GoHAP1-Cef (**c**).

When examining the antibacterial effect by means of the broth dilution method, the PEEK-HAP1-Cef sample possessed a higher antibacterial effect against *S. aureus* compared to PEEK-HAP1 and the control sample (PEEK only; Figure 9A). The OD recorded for PEEK-HAP1-Cef was close to zero with linear performance over 168 h, whereas a high OD was detected for PEEK-HAP1 and control. The efficient killing of bacteria over 168 h is probably related to the amount of drug loaded (~1 mg/cm^2^) and the release effect with an initial burst effect followed by a stable release up to 150 h. These results indicate that PEEK with a GoHAP layer loaded with Cef prevents *S. aureus* over a long period of time (i.e., 168 h), which is required for antibacterial implants. There seems to be a good relationship between the loaded amount, release effect, and antibacterial activity. Photos of the tested tubes are presented in Figure 9B, showing that the presence of Cef led to a clear picture (no observed bacterial growth) compared to PEEK-HAP1 and control samples. This observation confirms the results obtained with the broth dilution method.

**Figure 10:**
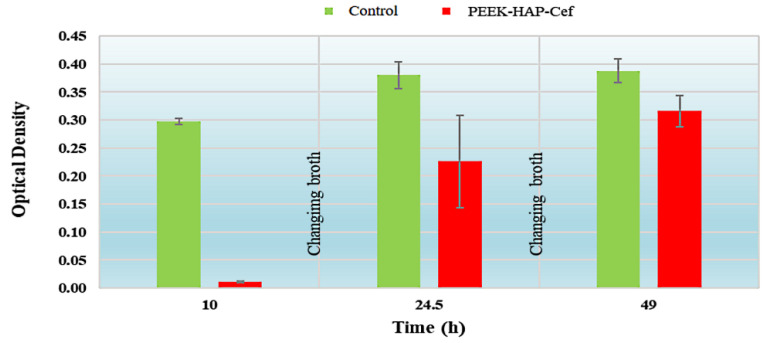
The antibacterial activity against *Staphylococcus aureus* ATCC 25923 by means of the broth dilution method (with change of broth medium) after incubation of up to 49 PEEK-GoHAP1-Cef compared to control. Data are presented as mean ± SD.

For further evaluation of the antibacterial properties, we used the broth dilution method to change the broth from the samples and supply a new volume of broth. Removing the broth should remove the drug because it is a water-soluble compound. For this reason, we changed the broth two times after incubation for 10 and 24.5 h (Figure 10). PEEK-HAP1-Cef had a lower OD compared to the control. By changing the broth at 10 h and 24.5 h, the OD increased, especially at 25.5 h compared to 10 h, compared to the OD before changing the broth. Interestingly, these results disclose that Cef is attached to the surface as well as in the pores of GoHAP.

Overall, the results obtained in antibacterial evaluation studies have demonstrated that PEEK-HAP-Cef coatings not only facilitate a short-term antibacterial effect, but also promote protection from *S. aureus*. Bone growth could be promoted because a small fraction of drug is present in the GoHAP layer and more on the surface. Our results are in line with earlier studies highlighting the importance of the antibacterial effects of HAP coatings [17,82,83,84].

Coating medical implants with HAP nanoparticles using sonocoating has many advantages. This fabrication method does not affect the structure and crystallinity of the nanoHAP because the coating temperature is low. The same factor is responsible for the layer possessing a highly active surface and nanoporosity, allowing a relatively large amount of drug to be loaded per unit area.

## 4. Conclusions

The sonocoating technique achieves a layer of nano-hydroxyapatite GoHAP on the surface of PEEK via a facile process. The technique allows the thickness to be controlled in the range of 200 to 760 nm. The sonocoated material is strongly hydrophilic. The GoHAP layer has a highly developed specific surface that favors drug absorption. Absorption of Cef antibiotics of up to 1 mg/cm^2^ was achieved. The amount of drug loaded can be controlled by the type of GoHAP, the thickness and porosity of the layer, and the concentration of the drug. An initial burst of 86 to 96% of the drug being released within 24 h is followed by a linear, stable release. The PEEK-HAP1-Cef coating has a sufficient antibacterial effect against *S. aureus*, as shown by zone inhibition and broth dilution. Thus, our results could pave the way for antibacterial coatings on medical implants.

## Figures and Tables

**Table 1:** GoHAP coating thickness on a PEEK substrate after several iterations.

	GoHAP1 1st Coating	GoHAP1 2nd Coating	GoHAP1 3rd Coating	GoHAP1 5th Coating
Thickness of layer (nm)	218 ± 33	368 ± 17	462 ± 30	478 ± 20
	**GoHAP3 1st Coating**	**GoHAP3 2nd Coating**	**GoHAP3 3rd Coating**	**GoHAP3 5th Coating**
Thickness of layer (nm)	246 ± 29	340 ± 40	368 ± 32	763 ± 52

Values are given as mean ± SD.

**Table 2:** Water contact angle for nanoHAP coating on PEEK substrates after several iterations.

Sample Code	PEEK	HAP1 1st Coating	HAP1 2nd Coating	HAP1 3rd Coating	HAP1 5th Coating	HAP3 1st Coating	HAP3 2nd Coating	HAP3 3rd Coating	HAP3 5th Coating
Contact angle	90° ± 1°	69° ± 4°	69° ± 5°	55° ± 5°	64° ± 4°	52° ± 5°	55° ± 6°	51° ± 5°	54° ± 6°

Values are given as mean ± SD.

**Table 3:** Amount of Cef loaded in GoHAP-coated PEEK after five iterations.

GoHAP Type	Mean Drug ± SD (mg/Substrate 1 cm^2^)
PEEK-GoHAP1-Cef	1.09 ± 0.13
PEEK-GoHAP3-Cef	0.94 ± 0.16

No significant differences were found by means of least significance differences from one-way ANOVA.
